# The role of *CACNA1S *in predisposition to malignant hyperthermia

**DOI:** 10.1186/1471-2350-10-104

**Published:** 2009-10-13

**Authors:** Danielle Carpenter, Christopher Ringrose, Vincenzo Leo, Andrew Morris, Rachel L Robinson, P Jane Halsall, Philip M Hopkins, Marie-Anne Shaw

**Affiliations:** 1 MH Investigation Unit, Academic Unit of Anaesthesia, St James's University Hospital, Leeds, LS9 7TF, UK; 2 Wellcome Trust Centre for Human Genetics, Roosevelt Drive, Oxford, OX3 7BN, UK; 3 Institute of Integrative and Comparative Biology, LC Miall Building, Faculty of Biological Sciences, University of Leeds, Leeds, LS2 9JT, UK

## Abstract

**Background:**

Malignant hyperthermia (MH) is an inherited pharmacogenetic disorder of skeletal muscle, characterised by an elevated calcium release from the skeletal muscle sarcoplasmic reticulum. The dihydropyridine receptor (DHPR) plays an essential role in excitation-contraction coupling and calcium homeostasis in skeletal muscle. This study focuses on the gene *CACNA1S *which encodes the α1 subunit of the DHPR, in order to establish whether *CACNA1S *plays a major role in MH susceptibility in the UK.

**Methods:**

We investigate the *CACNA1S *locus in detail in 50 independent MH patients, the largest study to date, to identify novel variants that may predispose to disease and also to characterise the haplotype structure across *CACNA1S*.

**Results:**

We present *CACNA1S *cDNA sequencing data from 50 MH patients in whom *RYR1 *mutations have been excluded, and subsequent mutation screening analysis. Furthermore we present haplotype analysis of unphased *CACNA1S *SNPs to (1) assess *CACNA1S *haplotype frequency differences between susceptible MH cases and a European control group and (2) analyse population-based association via clustering of *CACNA1S *haplotypes based on disease risk.

**Conclusion:**

The study identified a single potentially pathogenic change in *CACNA1S *(p.Arg174Trp), and highlights that the haplotype structure across *CACNA1S *is diverse, with a high degree of variability.

## Background

Malignant hyperthermia (MH) is an inherited disorder of skeletal muscle, which predisposes to an increased release of calcium into the myoplasm under certain pharmacological conditions. Inhalational anaesthetics and the muscle relaxant suxamethonium can trigger an MH crisis and lead to acceleration of muscle metabolism and contractile activity generating heat and leading to hypoxaemia, metabolic acidosis, rhabdomyolysis and a rapid rise in body temperature. This condition is potentially fatal if not recognised and treated promptly.

Biochemical studies have shown that an MH crisis is due to an abnormal cellular calcium homeostasis within the skeletal muscle [1]. Within skeletal muscle the sarcoplasmic reticulum (SR) controls the process of Ca^2+ ^release, playing a major role in the process of excitation-contraction (E-C) coupling. During E-C coupling depolarisation of the sarcolemma initiates a conformational change in the voltage-gated Ca^2+ ^channel (dihydropyridine receptor (DHPR)) subsequently activating the Ca^2+ ^release channel (Ryanodine receptor (RyR1)) to release Ca^2+ ^from the SR [2]. During an MH crisis an elevated rate of cellular Ca^2+ ^release from the SR is observed due, in part, to a reduced activation and increased deactivation threshold of the RyR1 [3], or from uncoupling of the DHPR-RyR1 interaction [4].

Genetic analyses have demonstrated that MH susceptibility exhibits locus heterogeneity, with significant observations for linkage to chromosome 1q [5,6] and 19q [7,8]. The locus on chromosome 19q has been identified as the gene encoding the skeletal muscle ryanodine receptor (*RYR1*) [8], and that on chromosome 1q as the gene encoding the α1 subunit of the DHPR (*CACNA1S*) [5]. There is a finely balanced interaction between the gene products of *RYR1 *and *CACNA1S*, which are only beginning to be understood with alterations in both gene products affecting E-C coupling and modifying Ca^2+ ^regulation [3,4].

Much research into MH susceptibility has been focused on the *RYR1 *locus and it is recognised that *RYR1 *plays a major role in susceptibility to MH. There are now over 178 mis-sense mutations described across *RYR1 *that co-segregate with MH susceptibility, 29 of which have been functionally characterised and are used diagnostically (reviewed in [9]). In the UK, *RYR1 *plays a part in MH susceptibility in over 70% (394/554) of UK pedigrees. Considerably less, however, is known about *CACNA1S*. Previous studies have demonstrated linkage to chromosome 1q within MH families that show *RYR1 *exclusion [10], but to date there is only a single mis-sense change (p.Arg1086His) described in *CACNA1S *in association with MH [5]. This change was first detected in a single extended French family in 12 individuals all diagnosed as susceptible to MH, and absent from the 6 individuals diagnosed as normal [5]. In a North American study of 98 independent MH samples this change was also identified in a single family [11], in 2 from the 5 MH diagnosed individuals. p.Arg1086His was not detected in 100 independent normal French chromosomes [5], nor in 150 unrelated North American normal samples [11]. Interestingly, this change has further been described alongside an *RYR1 *alteration (p.Pro4973Leu) in a single individual [12], where the rest of the family diagnosed as MHS were accounted for by either the *RYR1 *change (three individuals) or the *CACNA1S *change (two individuals), suggesting a potentially more complex means of MH susceptibility involving multiple gene products.

The aim of this study is to investigate the *CACNA1S *locus in detail and to determine whether *CACNA1S *may play a major role in MH susceptibility in the UK. As targeted sequencing for *RYR1 *has led to potential bias in mutation detection, we have sequenced the full cDNA transcript of *CACNA1S *for novel changes in 50 independent MH patients. We report here the findings of this sequencing and subsequent mutation screening. Furthermore, characterisation of the haplotype structure across *CACNA1S *is investigated. We present analysis using unphased *CACNA1S *SNP data directly to (1) assess *CACNA1S *haplotype frequency differences between MH susceptible cases and a population control group and (2) to analyse population-based association via clustering of *CACNA1S *haplotypes based on disease risk.

## Methods

### *In-vitro *contracture testing

There is a well defined and standardised protocol for the laboratory confirmation of suspected MH cases and testing of family members. The *in vitro *contracture test (IVCT) involves exposure of skeletal muscle biopsy specimens to incremental concentrations of halothane or caffeine in an irrigated tissue bath and the subsequent measurement of muscle contracture in response to the applied stimulants. All individuals were phenotyped by the IVCT according to the European MH Group (EMHG) guidelines  at the MH Investigation Unit at St James's Hospital, Leeds, UK. The European protocol assigns the patient to one of three laboratory diagnostic categories, MHS, MHN or MHE according to whether their muscle displays increased sensitivity to both, none or only one of the stimulants respectively. Both MHS and MHE categories are deemed to represent clinical susceptibility to MH.

### Samples

This study utilises the largest worldwide resource of genotyped MH samples from patients phenotyped in a single diagnostic centre. *CACNA1S *sequencing was performed on 50 independent UK MH susceptible samples, with approval by the Leeds East Local Research Ethics Committee and written informed consent from all patients. These samples comprise 30 MHS samples and 20 MHE (responding to halothane but not caffeine) samples; 14 of these MHE samples are from probands who suffered a clinical reaction. The remaining 6 MHE samples are the only available representative from these individual families. However, a recent study by our group, using transmission data, supports the classification of MHE samples as affecteds [13]. All 50 of these samples have had the *RYR1 *cDNA transcript sequenced and have had no variants detected. There are 30 males and 20 females in the cohort and the ages range from 10-75 years, with a mean of 33 years. The IVCT data for the 50 patients show a median (range) contracture of 0.6 g (0.2 - 4.7 g) at 2% halothane and 0.2 g (0 - 3.6 g) at 2 mM caffeine.

Mutation screening was performed on an additional 410 independent UK MH patients to give a total of 460 independent MH patients represented. Of the 460 independent MH patients, 340 (74%) have an *RYR1 *mis-sense change assigned; of these, 298 (65%) co-segregate with disease, 226 (49%) have a functionally characterised *RYR1 *change and there are 8 instances of compound heterozygosity with two different *RYR1 *changes (one instance each of c.1021G>A/c.7025A>G, c.7063C>T/c.7025A>G, c.7036G>A/c.14817C>A, c.4024A>G/c.4088C>T, c.5441T>A/c.7528T>C and c.10616G>A/c.14210G>A and two instances of 7300G>A/7373G>A). 100 independent MHN samples were also screened.

*CACNA1S *haplotype analysis was performed on 460 independent UK MH patients, the same as used for mutation screening assays. Population control samples (480) for the haplotype analysis were obtained from a DNA panel of Human Random Controls manufactured by the European Collection of Cell Cultures (ECACC). The DNA is derived from peripheral blood lymphocytes of UK Caucasian donors with informed consent.

### *CACNA1S *sequencing

*CACNA1S *cDNA sequencing was performed on 50 independent MH susceptible individuals, who did not have an *RYR1 *mis-sense change after having previously been sequenced for the *RYR1 *cDNA transcript. cDNA prepared from total RNA isolated from muscle biopsy specimens was used to sequence the ~6.16 kb *CACNA1S *cDNA, using 12 overlapping fragments of approximately 700 bp in length, read in both the forward and reverse direction and analysed on an ABI3730.

### Mutation analysis

A novel p.Arg174Trp/c.520C>T change in exon 4 causes a loss of an *Msp*I site, thus further screening analysis was performed on genomic DNA using the forward primer 5'-CTC AAG CAT GGA CAG GAC AC-3' and reverse primer 5'-AGG AAG GGA GAG GAG AAA GG-3' to generated an amplicon of 279 bp. In the normal (c.520*C) this is cleaved to produce 3 fragments of 49 bp, 67 bp and 163 bp. Cleavage at one of the sites fails to occur in the presence of the mutated allele, c.520*T, thus generating 2 fragments of 116 bp and 163 bp in length.

The previously described *CACNA1S *mutation p.Arg1086His/c.3257G>A in exon 26 was screened for in the full cohort of independent UK MH patients using an assay developed in-house as follows: forward 5' ATG CAC CCT ACC CTA TCT CC-3' and reverse 5'-GGA GCA GGG AGC CTA GTT AC-3' primers generate an amplicon of 998 bp in length. In the normal (c.3257*G) this is cleaved by *Hha*I to produce 3 fragments of 362 bp, 316 bp and 313 bp. Cleavage at one of the sites fails to occur in the presence of the mutated allele, c.3257*A, generating 2 fragments of 629 bp and 362 bp in length.

### Haplotype analysis

#### Haplotype construction

Focusing at the genomic DNA level there are >175 SNPs described across the 73 kb *CACNA1S *gene; predominantly listed in internet databases sources, in particular the CEPH population of the Hap-Map project , and a further 3 identified through in-house *CACNA1S *sequencing. When concentrating on SNPs with a minor allele frequency greater than 0.05 the total number of described SNPs spanning the gene is reduced to 115, 16 of which are located in exons. Using the Tagger software on Haploview we selected eight informative SNPs to span *CACNA1S*. The final list of SNPs chosen is detailed in Table 1.

**Table 1:** Details of the SNPs selected for inclusion in the *CACNA1S *haplotype analysis

**NCBI reference**	**Location**^**a**^	**Chromosomal position**^**b**^	**Het**^**c**^	**ABI assay number**	**SNP number**
rs1546416	Intron42-43	199276179	0.468	C___2826986_1_	1

rs12029212	Intron32-33	199288242	0.303	C__25474094_10	2

rs7415038	p.Phe801	199305310	0.504	C___3135170_10	3

rs10920105	Intron15-16	199309241	0.468	C__25652884_10	4

rs9427714	p.Gly505	199313734	0.495	C__25652932_10	5

rs2296383	p.Ile199	199327488	0.528	C__15752541_10	6

rs1536129	Intron2-3	199335814	0.439	C___1942693_10	7

rs1325309	Intron1-2	199346081	0.414	C___1942703_20	8

^a^Location of each SNP according to reference sequence

^b^Chromosomal position according to NCBI reference sequence for chromosome 1

^c^Heterozygosity calculated from our data for the 480 population control samples

All SNPs were genotyped using Taqman^® ^methodology. For all SNPs there is an ABI-assay-on-demand available (Table 1). All allelic discrimination assays were carried out on an ABI 7900 according to the manufacturer's instructions. Linkage disequilibrium between the SNPs was calculated using Haploview software [14].

#### Statistical analysis

The program PHASE (version 2) was used to reconstruct haplotypes from the unphased *CACNA1S *genotype data and to perform case control permutation tests between MHS samples and population control samples [15,16]. PHASE calculates the posterior probability distribution of haplotypes through a Bayesian statistical approach, combining a specified prior for a statistical model for population genetics and likelihood information. The program has a function for case control permutation testing. This tests the null hypothesis that haplotype frequencies are the same in cases and controls, versus the alternative hypothesis that haplotype frequencies are different between the two groups.

The program GENEBPM, a program designed for use with candidate genes, tests for association of disease with causal variants at an unseen functional polymorphism [17,18]. This program makes use of the expectation that a pair of haplotypes carrying the same disease mutation are more likely to share a more recent common ancestry than a random pair of haplotypes in the population and thus are more likely to be similar to each other in terms of their allelic make-up at flanking markers. Furthermore, output of the algorithm can be used to ascertain clusters of haplotypes that are associated with specific causative variants, and to estimate the odds of disease for these unobserved alleles.

## Results

### *CACNA1S *sequencing

Full cDNA sequencing identified non-synonymous changes in *CACNA1S *in 12 individuals, 24% of the MH cases. Sequence changes lead to modifications of amino acids at positions 69 (n = 4), 174 (n = 1), 258 (n = 4), 458 (n = 13), 606 (n = 1), 1541 (n = 4) and 1660 (n = 5) where the number in parentheses represents the total number of MH patients with each change. All are present as heterozygous changes, except the change at position 458 which was also observed in both homozygous forms. However, the changes at positions 1541 and 1660 are previously described polymorphisms (rs3850625 and rs13374149 respectively). The change at position 458 has also been described as polymorphic [5] and indeed was found to be highly variable in our cohort with a heterozygosity of 0.425. The other substitutions detected were in codons determining amino acids that are conserved in rabbit, cat, mouse and zebrafish (NCBI reference sequences NP_001095190, NP_001033694, NP_001074492.1 and NP_999891.1 respectively) and are therefore potentially deleterious mutations rather than infrequent polymorphisms. However, the first change p.Ala69Gly, whilst being detected in 4 MH susceptible individuals, was also detected in 7 from 100 MHN samples, and is therefore likely to be a polymorphism. Furthermore, the changes p.Gly258Asp and p.Ser606Asn, whilst not being detected in 100 MHN controls, were observed to be frequently discordant with MH status in families. Within the 4 p.Gly258Asp families there are a total of 24 individuals, comprising 5 MHS, 7 MHE and 12 MHN samples, and the p.Gly258Asp change was observed in a total of 3 MHN samples, 2 MHS and 3 MHE samples, whilst the p.Ser606Asn was detected in both the MHE and MHN siblings within a single family. There was no example of compound heterozygosity with these rare changes. These changes can be added to the growing number of non-synonymous changes reported across *CACNA1S*, over half (10) of which are likely to be polymorphisms (see Table 2).

**Table 2:** Reported variants in the coding sequence of *CACNA1S*

**Reported mis-sense changes in *CACNA1S***						
**Exon**	**Nucleotide change**^**a**^	**Protein change**	**Incidence**^**b**^	**Population**	**Disease status**^**c**^	**References**

1	c.64A>T	p.Glu22Val	Unknown	Unknown	unknown	ss95806

2	c.205C>G	p.Ala69Gly	11	UK	polymorphism	This study (ss43973054)

4	c.520C>T	p.Arg174Trp	1	UK	MH	This study

6	c.773G>A	p.Gly258Asp	4	UK	polymorphism	This study

10	c.1373T>A	p.Leu458His	14	UK & France	polymorphism	This study, 5

11	c.1582C>G	p.Arg528Gly	1	China	HOKPP	[27]

11	c.1583G>A	p.Arg528His	77	Europe, USA, Japan, Korea & china	HOKPP	[28-43]

12	c.1669G>A	p.Arg557His	1	Unknown	unknown	ss6793785

12	c.1817G>A	p.Ser606Asn	1	UK	polymorphism	This study

21	c.2691G>T	p.Arg897Ser	1	France	HOKPP	[44]

26	c.3257G>A	p.Arg1086His	3	USA & France	MH	[5,11,12]

30	c.3684C>G	p.Arg1239Gly	10	USA & Korea	HOKPP	[28,33,42,45,46]

30	c.3685G>A	p.Arg1239His	50	USA, Europe & Japan	HOKPP	[29,33,34,39,42,45,47]

37	c.4475C>A	p.Ala1492Asp	1	Italy	polymorphism	[48]

38	c.4621C>T	p.Arg1541Cys	4	UK	polymorphism	This study (rs3850625)

40	c.4978G>A	p.Arg1660His	5	UK	polymorphism	This study (rs13374149)

44	c.5404T>C	p.Leu1802Ser	Unknown	UK	polymorphism	rs12139527

44	c.5525G>C	p.Glu1842Asp	Unknown	UK	polymorphism	rs1042379

^a^Numbering based on cDNA ref: ENST00000263942.

^b^Total number of independent observations.

^c^Disease status reported with variant; malignant hyperthermia (MH), and hypokalemic periodic paralysis (HOKPP).

The final mis-sense variation that was detected, p.Arg174Trp, was found in an MHS sample, was concordant with disease within the family and also not detected in 100 MHN control samples. The mother of the proband was diagnosed MHS through the IVCT, and also had the p.Arg174Trp alteration. A sibling of the proband, diagnosed normal through the IVCT, did not have the p.Arg174Trp change. The MH proband, in whom the p.Arg174Trp was detected, developed intense masseter muscle spasm and generalised muscle rigidity lasting 8 minutes after administration of the inhalation anaesthetics propofol, fentanyl and halothane and the muscle relaxant suxamethonium (1.5 mg/kg). Post-operatively there was severe muscle stiffness that persisted for 2 weeks and a peak serum creatine kinase concentration of 14,500 IU/L (normal < 220 IU/L). The IVCT results for this individual were 0.35 g contracture at 2% halothane and 0.2 g contracture at 2 mM caffeine: laboratory classification (EMHG) MHS.

The cDNA sequencing further identified 3 novel silent changes in the *CACNA1S *gene. These were located in 2 different exons; p.Leu766/c.2296C>T minor allele frequency of 0.01 and a heterozygosity of 0.02, and p.Ile781/c.2343C>T, with a minor allele frequency of 0.031 and a heterozygosity of 0.06 were located in exon 17 and p.Pro1622/c.4866C>T with a minor allele frequency of 0.208 and a heterozygosity of 0.33 was located in exon 40. There is no significant linkage disequilibrium detected between the markers, with r^2 ^= 0 between 4866*C and both 2296*C and 2343*C, and r^2 ^= 0.32 between the neighbouring markers 2296*C and 2343*C. Linkage disequilibrium was also not detected between these markers and their adjacent markers across *CACNA1S*, i.e. between 4866*C with either rs3850625*C or rs13374149*G and nor between 2296*C and 2343*C with rs7415038*T or rs1684767*C.

### Mutation screening

The p.Arg174Trp change was identified in a single family showing full concordance with disease status and not identified in 100 normal controls. Accordingly we screened for the presence of this site in the full UK cohort of 410 independent MH families. The p.Arg174Trp change was not detected in any other UK family.

The previously described *CACNA1S *mutation p.Arg1086His in exon 26 was screened for in the 460 independent UK MH patients. This change was not detected in any UK MH family, nor the 100 MHN controls.

### Haplotype analysis

A total of 460 independent UK MH patients and 480 Caucasian population controls were typed for all eight *CACNA1S *SNP markers. These 8 SNPs were used to reconstruct haplotypes from the unphased data using PHASE and GENEBPM. From GENEBPM there were a total of 23 haplotypes with an estimated population frequency ≥0.01 (1%), and these are detailed in Table 3, along with a breakdown of the haplotype frequencies for each study group (MHS, PC and also the subset of 50 samples that were sequenced for *CACNA1S*) calculated using PHASE. The single SNPs were tested for association with disease using a Spearman rank correlation between MHS samples and PC. There was no evidence for significant associations with any of the markers except p.Ile199, where p = 0.014.

**Table 3:** Details of the 23 *CACNA1S *haplotypes with an estimated population frequency ≥ 0.01 in at least one group.

**Haplotype Name**	**rs1546416**	**rs12029212**	**rs7415038**	**rs10920105**	**rs9427714**	**rs2296383**	**rs1536129**	**rs1325309**	**Estimated haplotype frequencies** **(Std Error)**^**a**^		
									
									**MHS**	**PC**	**50 sequenced samples**
H1	2	2	1	1	1	1	1	1	0.084 (0.008)	0.081 (0.007)	0.081 (0.018)

H2	1	2	1	1	1	1	1	1	0.047 (0.007)	0.075 (0.006)	0.055 (0.018)

H3	2	2	2	1	2	2	1	1	0.065 (0.007)	0.059 (0.007)	0.044 (0.014)

H4	2	2	2	2	2	2	2	1	0.05 (0.006)	0.046 (0.006)	0.07 (0.018)

H5	2	1	1	1	1	1	1	1	0.043 (0.008)	0.04 (0.005)	0.099 (0.019)

H6	2	2	2	1	2	2	2	2	0.03 (0.005)	0.05 (0.005)	0.016 (0.009)

H7	1	2	1	1	1	2	2	1	0.03 (0.005)	0.04 (0.004)	0.012 (0.01)

H8	2	2	2	1	2	2	1	2	0.035 (0.005)	0.032 (0.007)	0.035 (0.009)

H9	2	2	2	2	2	1	1	1	0.031 (0.005)	0.036 (0.004)	0.017 (0.012)

H10	2	2	1	1	1	1	1	2	0.036 (0.006)	0.027 (0.005)	0.03 (0.012)

H11	2	2	2	1	2	2	2	1	0.026 (0.005)	0.029 (0.004)	0.029 (0.011)

H12	1	2	1	1	1	1	1	2	0.017 (0.004)	0.032 (0.005)	0.03 (0.012)

H13	1	2	2	1	2	2	1	1	0.026 (0.006)	0.018 (0.003)	0.03 (0.013)

H14	1	2	1	2	2	2	1	2	0.029 (0.004)	0.014 (0.003)	0.058 (0.01)

H15	2	2	2	2	2	2	2	2	0.025 (0.005)	0.02 (0.005)	0.029 (0.015)

H16	1	2	2	2	2	2	2	1	0.02 (0.006)	0.014 (0.004)	0.03 (0.014)

H17	2	2	2	2	2	1	1	2	0.021 (0.005)	0.019 (0.004)	0.015 (0.01)

H18	1	2	1	1	1	2	2	2	0.018 (0.005)	0.017 (0.003)	0.01 (0.009)

H19	2	2	2	2	2	2	1	1	0.017 (0.004)	0.018 (0.003)	0.005 (0.006)

H20	2	1	1	1	1	1	1	2	0.006 (0.003)	0.025 (0.006)	0.026 (0.013)

H21	1	1	2	2	2	1	1	1	0.015 (0.004)	0.01 (0.002)	0.006 (0.007)

H22	1	2	2	2	2	2	1	2	0.017 (0.004)	0.006 (0.002)	0.005 (0.007)

H23	2	2	2	2	2	2	1	2	0.019 (0.004)	0.007 (0.003)	0.015 (0.01)

^a^The estimated haplotype frequencies and standard errors (Std Error) were calculated separately for each of the MHS, Population Control and 50 cDNA *CACNA1S *sequenced samples using the program PHASE.

Case control permutation testing was performed between MHS samples and the population control samples to test for differences in *CACNA1S *haplotype frequencies using the program PHASE. There was a small but significant difference observed with this comparison (p = 0.02), providing evidence for association between MH and *CACNA1S*. However, for the same comparison using the GENEBPM program to analysis haplotype relative risk of disease, there was no categorical evidence of *CACNA1S *haplotype association with MH (posterior probability  = 0.46).

**Figure 1:**
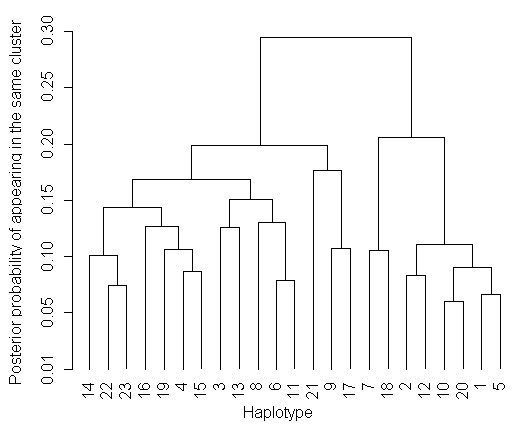
**Dendrogram of the 23 marker haplotypes with estimated population frequency ≥0.01 (1%), to demonstrate similarities between haplotypes in terms of disease risk and marker sharing created from the output of a single run of the MCMC algorithm in GENEBPM**. Haplotypes are coded according to their relative frequency, thus haplotype 1 is the most common haplotype, and haplotype 23 the least.

To illustrate the posterior similarities between haplotypes in terms of their risk of carrying causal variants and allelic make-up, a dendrogram can be constructed. Figure 1 presents a dendrogram of the 23 *CACNA1S *haplotypes with estimated relative frequency ≥1% from the analysis of MHS cases versus population controls. These 23 haplotypes are coded according to their relative frequency, where 1 represents the most frequent haplotype and 23 the least frequent. The dendrogram shows considerable posterior similarity between haplotypes and demonstrates no apparent clustering of haplotypes, suggesting that there is no clear high risk disease variant. Additionally, the haplotype analysis reveals that there are numerous relatively rare haplotypes (there are only three haplotypes with a frequency >5% in population controls), suggestive of an elevated degree of haplotype diversity potentially resulting from a high rate of recombination across the locus and a low level of linkage disequilibrium.

To exclude any influence of *RYR1 *on the analysis, a case control permutation test in PHASE was also performed between the 480 population control samples and the 50 samples that have been cDNA sequenced for *CACNA1S*. There was no overall significant difference in *CACNA1S *haplotype frequency observed between these groups. The haplotypes H5 and H14 were observed at a noticeably higher frequency in the 50 sequenced samples (0.099 and 0.058 respectively) than the population controls (0.043 and 0.029 respectively) (see Table 3), however given that the haplotype frequencies are relatively small these observations are unlikely to be significant. Further analysis with GENEBPM also provided no categorical evidence of *CACNA1S *haplotype association with MH (posterior probability  = 0.415), suggesting that *CACNA1S *does not play a major role in MH susceptibility, however due to the small number in the cDNA sequenced group this comparison may lack power.

Furthermore, to investigate whether there were any differences in *CACNA1S *haplotype frequencies between MH phenotypes, an additional case control permutation test was performed on the subset of 50 samples that underwent cDNA sequencing between the MHS (n = 30) and MHE (n = 20) samples using all 8 SNPs. There was no significant difference in *CACNA1S *haplotype frequency observed between the MHS and MHE samples (p = 0.27); however, again due to the small numbers in each group this comparison may lack power.

## Discussion

As more is being understood about the nature of susceptibility to MH it is becoming increasingly apparent that it is complex and cannot always be simply described as autosomal dominant. There is evidence for variation in clinical severity and IVCT phenotype, resulting from the same mis-sense change in *RYR1 *[19,20]. There are also reports of compound heterozygotes in *RYR1 *[[12,21], unpublished UK observations] and an individual with mutations in both *RYR1 *and *CACNA1S *[12]. Furthermore, we have previously demonstrated, using transmission disequilibrium testing, that multiple interacting gene products affect susceptibility to MH [10,22]. Even within families that showed linkage to *RYR1 *evidence has been provided for linkage to other loci elsewhere in the genome [10].

This study focused on *CACNA1S *encoding the α1 subunit of the DHPR. In the largest study to date, of 50 MH patients, we identified a single, potentially pathogenic, variant p.Arg174Trp. The p.Arg174Trp change is situated at a site that is conserved in rabbit, cat, mouse, and zebrafish and causes a change in the charge of the amino acid from basic to non-polar. The amino acid in question lies in the S4 segment domain of the DHPR thought to function as a voltage sensor, thus a change in charge may alter the voltage sensor mechanism and consequently disrupt the cellular calcium homoeostasis. Further functional work to support these observations would be valuable.

This work also identified two other variants (p.Gly258Asp, p.Ser606Asn) thought to be polymorphic as they do not show disease concordancy, but which were not present in control chromosomes. Whilst it is likely that they are indeed polymorphisms, the fact that they lie in conserved regions of the protein and cause a change in the polarity of the amino acid substituted suggests otherwise and there is the potential that they could play a minor role in, or have a modifier effect on, disease phenotype. Since there is evidence that MH may not necessarily be a simple single gene disorder, there is the possibility that both of these changes are present together with an additional major change and in some instances account for discordancy with disease; i.e. these mutations may be necessary, but not sufficient, to cause MH susceptibility in particular individuals.

Comparison of *CACNA1S *haplotype frequencies between susceptible cases and UK Caucasian population controls identified no significant haplotype frequency differences. Even given that this is the largest standardised and genotyped MH database worldwide, there are a limited number of MH patients, which could reduce the power to detect a significant association between *CACNA1S *haplotype with MH. However, the haplotype analysis does provide some evidence for an elevated haplotype diversity, potentially resulting from the high rate of recombination observed across the locus and a low level of linkage disequilibrium detected, as seen in the present study and consistent with that observed in the HapMap project. Coupling this observation with the now growing number of reported non-pathogenic non-synonymous changes described across *CACNA1S*, it is possible that this locus can tolerate a high degree of variability. This variability could affect the conformation of the DHPR protein, which has implications not only in the E-C coupling of skeletal muscle but also in that of cardiac muscle.

Our data suggest that, whilst *CACNA1S *may play a role in MH manifestation in the UK, it is not a major locus, thereby suggesting that there are other loci with importance in MH susceptibility. As well as the reported linkage to chromosome 1q and 19q there are alternative loci proposed on chromosomes 7q [23] and 17q [24], however no contributory mutations have been identified in these regions. It is highly probable that any novel genes for MH susceptibility will play a minor role. An alternative method to identify genes responsible for MH could be to take a candidate gene approach and focus on genes whose products are directly involved with E-C coupling and Ca^2+ ^regulation, for example the other subunits of the DHPR, calmodulin [25]and JP-45 [26].

## Conclusion

We have previously proposed that several independent genes can influence the MH phenotype [10,22]. Here we have presented evidence for a novel variant in *CACNA1S *which could have the potential to directly influence MH susceptibility. There was also a possible indirect or modifier effect of *CACNA1S *in a small number of families, likely to cause MH in combination with another, as yet unknown, locus. The evidence for the existence of multiple independent loci that influence MH susceptibility is now increasing and the disorder appears to be more complex than previously thought. To fully comprehend MH and all the gene product interactions we need to identify and characterise all the multiple loci involved.

## Competing interests

The authors declare that they have no competing interests.

## Authors' contributions

DC participated in the design of the study, carried out the Taqman^® ^genotyping, haplotype analysis and drafted the manuscript. CR and VL carried out the sequencing, screened for the changes Arg1086His and Arg174Trp and performed family studies. AM provided assistance with haplotype analysis. RLR participated in the design of the study. PJH and PMH carried out and determined the IVCT phenotypes. PMH and MAS designed the study, coordinated the study, and participated in the analysis and manuscript preparation. All authors approved the manuscript.

## Pre-publication history

The pre-publication history for this paper can be accessed here:


